# A comparison of three dose timings of methylprednisolone in infant cardiopulmonary bypass

**DOI:** 10.1186/2193-1801-3-484

**Published:** 2014-08-29

**Authors:** Davinia E Withington, Patricia S Fontela, Karen P Harrington, Christo Tchervenkov, Larry C Lands

**Affiliations:** Department of Pediatric Anesthesia, McGill University Health Center/Montreal Children’s Hospital, 2300 Tupper Street, Room C-1118, Montreal, Quebec Canada; Department of Pediatrics, McGill University Health Center/Montreal Children’s Hospital, Montreal, Canada; Department of Epidemiology, Biostatistics, and Occupational Health, McGill University Montreal, Montreal, Canada; Department of Critical Care, Centre Hospitalier Universitaire Ste Justine, Montreal, Canada; Division of Pediatric Cardiothoracic Surgery, McGill University Health Center/Montreal Children’s Hospital, Montreal, Canada

**Keywords:** Cardiopulmonary bypass, Pediatric, Methylprednisolone, Inflammatory response, Leukotrienes, Oxidative stress, Oxygenation

## Abstract

Although commonly used in pediatric cardiopulmonary bypass (CPB) optimal dose and timing of steroid administration is unclear. We hypothesized that early administration of a commonly used dose of methylprednisolone given the evening before surgery (ultra-early) would be more effective in decreasing CPB-related inflammatory response than when given at induction of anesthesia (early) or in pump prime (standard).

This was a triple-arm, parallel, active control, superiority RCT including 54 infants <2 years old who were randomised to receive 30 mg/kg methylprednisolone at one of the 3 time points. Outcomes included alveolar-arterial oxygen gradient (AaDO2) during, 24, 48 and 72 hours post-CPB, IL-6, IL-8 and reduced (GSH) to oxidized (GSSG) glutathione ratio (pre-ultrafiltration on CPB, end-CPB and 24 hours), PICU length of stay (LOS) and ventilator days. Data were analysed using descriptive statistics and a random effects regression model.

The ultra-early group had higher Risk Adjusted Congenital Heart Surgery Score, lower age and longer CPB times than the other groups. No significant differences in AaDO2, IL-8, PICU LOS and ventilator days were observed between groups. Compared to the ultra-early group, the overall rise in IL-6 in the early and standard groups was lower, -27.8 pg/ml (95% CI -52.7,-2.9) and -35.3 pg/ml (95% CI -64.3,-6.34), respectively. GSH:GSSG was significantly lower in the standard group (-35.9; 95% CI -63.31,-8.5) at 24 hours post-CPB.

Ultra-early administration of methylprednisolone does not improve AaDO2 post-CPB, nor diminish cytokine release. Lower GSH:GSSG in the standard group suggests less oxidative stress. However despite statistical adjustments conclusions are limited by the unbalanced randomisation of the groups.

## Background

The use of corticosteroids as a means of diminishing the inflammatory response to cardiopulmonary bypass (CPB) has become a standard of practice in many institutions (Jonas 2004). Administration regimens vary since clear evidence of benefit or superiority of a particular timing remains elusive. However the administration of one dose of corticosteroid in pump prime is a standard of practice at many centres and is the preferred timing described in many studies (Hauser et al. 1998). In a survey of 36 international centres 97% of respondents used steroids in some cases and of these 83% used a single dose (Checcia et al. 2005). Since corticosteroids require approximately 2 hours for RNA transcription, and longer for maximal effects, administration in the pump prime may be too late to reduce the inflammatory response triggered by exposure to extracorporeal circulation (Ito et al. 1992; Xu et al. 1995).

Lodge et al. (1999) demonstrated improved alveolar-arterial oxygen gradient (A-aDO_2_) and total lung water in neonatal piglets given methylprednisolone 8 hours before CPB plus in the prime compared to prime alone. Two studies performed in humans investigated the effect of two doses of corticosteroids on CPB-induced inflammatory response, one of them given pre-operatively (Schroeder et al. 2003; Graham 2011). However, it remains unclear if the benefits observed in these studies are due to early administration of corticosteroids or use of two doses. Therefore, we performed this study with the objective of testing the hypothesis that a single ultra-early dose of methylprednisolone (the evening preceding surgery (Clarizia et al. 2011)) would improve A-aDO2 and other clinical and biochemical outcome measures compared to early (at anesthesic induction, 1–2 hours pre-CPB) *or* standard (during CPB) administration in children aged less than 2 years undergoing corrective congenital cardiac surgery.

## Methods

With approval from the Institutional Review Board of Montreal Children’s Hospital (MCH) we conducted a triple-arm, parallel, active control, superiority randomized controlled trial (RCT) with allocation ratio 1:1:1. MCH is a tertiary care university-affiliated hospital undertaking around 100 cardiac surgery cases requiring CPB/year.

### Patients and protocol

Patients under two years old scheduled for corrective cardiac surgery via median sternotomy, requiring CPB with moderate hypothermia, were considered eligible. Exclusion criteria were use of systemic corticosteroids within 3 months of enrolment, pre-existing primary pulmonary or renal pathology, surgery estimated as requiring <90 minutes CPB, e.g. secundum atrial septal defect, plan for deep hypothermia and/or circulatory arrest. One cardiac surgeon operated on all patients. Informed consent was obtained from parents at the pre-operative visit.

All patients received intravenous methylprednisolone 30 mg/kg as per institutional practice: ultra-early group (UE) the evening before CPB; early group (E) at anesthesia induction; standard group (S) in the pump prime. Timing of ultra-early doses conformed to known pharmacodynamics of corticosteroids (Ito et al. 1992; Xu et al. 1995). All patients received three intravenous solutions of identical appearance, one at each time point, two being normal saline and one methylprednisolone.

Patients were assigned to treatment group by simple randomization, with ratio 1:1:1, using computer-generated random numbers. MCH Pharmacy held the treatment allocation list and prepared identical appearing study medications with labeling “Dose 1, 2 or 3” to maintain blinding. All clinicians and research team members were blinded to the patient’s study group.

Intravenous induction was employed if an intravenous cannula was in place otherwise inhalational induction with sevoflurane preceded intravenous cannula insertion. Fentanyl or sufentanil plus pancuronium or rocuronium were used to facilitate intubation then maintenance was with infusions of sufentanil or fentanyl with midazolam plus pancuronium for neuromuscular blockade. Standard continuous monitoring was employed including intra-arterial blood pressure and central venous pressure.

Intravenous cefazolin was administered as antibiotic prophylaxis. In cases where the risk of intra- and post-operative bleeding was assessed as high, aprotinin or tranexamic acid were given by infusion throughout the case. All drugs received were noted in the database.

Prior to CPB heparin 400U/kg was given to achieve an ACT of >400 seconds. Pump prime was with irradiated reconstituted whole blood in infants <15 kg and with clear prime in those ≥15 kg. Dideco Lilliput™ oxygenators were used for infants up to 15 kg and D-905 (Dideco, Markham, Ontario) oxygenators for those >15 kg. At initiation of CPB furosemide 0.1 mg/kg and phentolamine 0.1 mg/kg were given plus the third syringe of study drug. The target hematocrit was 20%. Pump flow was maintained at 2–3 L/min/m^2^ during normothermia with reduction during cooling as per standard protocol. After aortic cross-clamping, cold cardioplegia (St Thomas’ Hospital solution) was infused for myocardial protection. Ultrafiltration was continuous throughout CPB.

After rewarming, inotropic support was introduced as necessary prior to separation from CPB. Heparin was reversed 1:1 with protamine sulphate. Blood products were used as needed to maintain a hematocrit of 40% and to control bleeding.

### Outcomes and data collection

The primary outcome was the A-aDO_2_ 72 hours after CPB. Our secondary clinical outcomes were 24-, 48-, and 72-hour fluid balance, weight, vasoactive inotrope score (Gaies et al. 2010), and urinary output, duration of mechanical ventilation and PICU length of stay (LOS). Secondary biochemical outcomes were plasma interleukin 6 and 8 (IL-6 and IL-8), and reduced and oxidized glutathione (GSH:GSSG) ratio.

Data were collected by trained research team members. Demographic (age, gender) and clinical information (weight, height, congenital cardiac malformation, CPB and cross-clamp time, minimal nasopharyngeal temperature during surgery, and Risk Adjusted Congenital Heart Surgery Score [RACHS]) were recorded for all participants.

Baseline blood samples for inflammatory response biomarkers were taken at the same time as routine pre-operative blood work. Further samples were taken during CPB (before ultrafiltration), immediately before and 24 hours after CPB cessation. Plasma for IL-6 and IL-8 assays was recovered immediately and frozen at -70°C before batch analysis using BD Biosciences™ ELISA kits according to the manufacturers’ instructions.

To evaluate oxidative stress we quantified total and oxidized glutathione in whole blood lysates. A low GSH:GSSG ratio is indicative of oxidative stress. For the measurement of total glutathione, 400 μl venous blood were deproteinized with 0.9% 5-sulfosalicylic acid. To measure GSSG, blood specimens were incubated with 9% 2-vinylpyridine to derivatize GSH prior to deproteinization. Deproteinized supernatants of whole blood were stored at -80°C pending analysis. To quantify glutathione (GSH), samples were diluted and neutralized with 100 mM phosphate buffer (pH 7.4) containing 5 mM EDTA. GSH analysis was conducted by the method established in our laboratory’s previous publications (Dauletbaev et al. 2001).

### Statistical methods

Based on the available evidence of the possible effect of methylprednisolone on A-aDO_2_ we defined an A-aDO_2_ difference, at 72 hours post-CPB, of 60 mmHg between groups (control and intervention) as clinically significant (Lodge et al. 1999). According to our calculation a sample size of 15 patients per group (total 45 patients) was sufficient to detect these effects at the 2-sided α level of 0.05 and with a power of 80%. We recruited a total of 54 patients to account for dropout.

Data were analyzed using an intention-to-treat approach. We used descriptive statistics, including mean (standard deviation – SD) and median (interquartile range – IQR), as well as frequency distribution, to describe the characteristics of the study groups. Differences in continuous variables were assessed by Student *t*-test; in the case of violation of the normality assumption (non-parametric data), the Mann–Whitney test was used. P values ≤ 0.05 were considered significant.

Due to the imbalance of RACHS score, CPB and cross-clamp time between groups, the data were also analysed using multivariate logistic and multivariate random effects regression models, which adjusted our results for possible confounding. This also addressed the fact that each patient had repeated measurements of the different variables. Statistical analysis was performed using R version 2.11.0.

## Results

**Figure 1:**
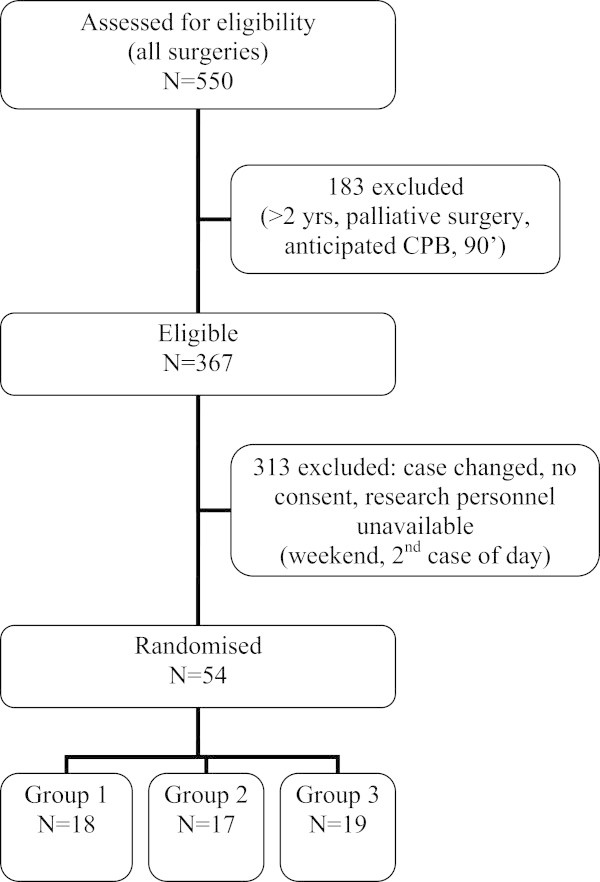
**Consort diagram.**

A total of 54 patients were recruited between 27th June 2001 and 24th April 2007. Participant flow is described in Figure 1*.* There were 2 protocol violations: 1) methylprednisolone 30 mg/kg was given 8 hours before surgery instead of study dose 1; 2) methylprednisolone 30 mg/kg was given at induction instead of study dose 2.

Demographic data are presented in Table 1. UE group patients were significantly younger but not smaller than those in the other groups. Although not statistically significant there was a trend to longer CPB and cross-clamp times in the UE group. Due to intraoperative changes in surgical plans, 17 (32%) infants had low flow or hypothermic circulatory arrest. Aprotinin was administered to 8 cases (UE group: 4, E group: 2, S group: 2).

**Table 1:** **Patient characteristics stratified by study group**

Variable	Ultra-early group (n = 18)	Early group (n = 17)	Standard group (n = 19)
**Males (%)**	10 (56)	12 (71)	12 (63)
**Age (months)**			
Mean (SD)	0.76 (0.86)	1.29 (2.44)	2.46 (3.68)
Median (IQR)	0.47 (0.26–0.91)	0.33 (0.27–1.00)	1.00 (0.27–3.97)
**Height (cm, mean, SD)**	51.9 (3.12)	52.4 (7.68)	55.9 (10.46)
**Pre-surgical weight (kg)**			
Mean (SD)	3.73 (0.84)	3.65 (1.30)	4.60 (2.27)
Median (IQR)	3.52 (3.16–3.96)	3.29 (2.94–3.89)	3.96 (3.58–5.02)
**Z score (mean)**			
Weight for length (SD)	-0.31 (1.60)	-0.71 (2.01)	-0.57 (2.05)
Weight for age (SD)	-0.72 (1.51)	-1.15 (1.29)	-1.53 (2.27)
**Cyanosis**	13 (72%)	12 (71%)	12 (63%)
**Lesion (%)**			
Tetralogy of Fallot	3 (17)	5 (29)	7 (37)
Transposition of great arteries	8 (44)	7 (41)	4 (21)
Ventricular septal defect	0	0	1 (5)
Atrioventricular canal	2 (11)	1 (6)	2 (11)
Hypoplastic Aorta	0	2 (12)	0
Other	5 (28)	2 (12)	5 (26)
**RACHS (mean, SD)**	3.00 (0.49)	3.06 (0.56)	2.53 (0.61)
**SatO**_**2**_ **RA (%, mean, SD)**	85.6 (8.29)	81.9 (13.18)	89.4 (8.76)
**CPB time (min)**			
Mean (SD)	231 (234)	256 (386)	155.4 (81)
Median (IQR)	169 (136–221)	172 (146–202)	133.0 (108–159)
**Aortic cross-clamp time (min)**			
Mean (SD)	98 (36)	97 (30)	81 (36)
Median (IQR)	88 (75–123)	97 (86–108)	75 (52–90)
**Nasopharyngeal temperature (°C, mean, SD)**	22.4 (3.47)	20.5 (2.93)	23.8 (3.78)
**Open chest (%)**	10 (56%)	10 (59%)	4 (26%)

SD = standard deviation; IQR = interquartile range); RACHS = Risk Adjusted Congenital Heart Surgery Score; SatO_2_ RA = oxygen saturation on room air; CPB = cardiopulmonary bypass.

**Figure 2:**
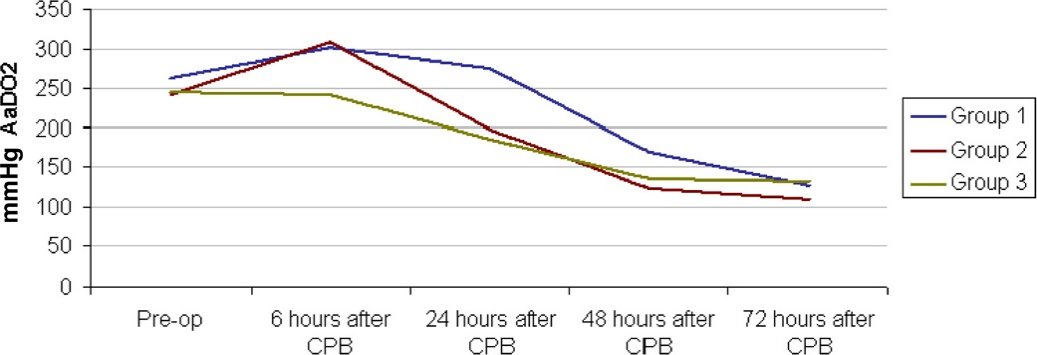
**Evolution of mean A-aDO**_**2**_ **over time stratified by study group.**

There was no significant difference in A-aDO_2_ between the groups at 72 hours post-CPB. Evolution of A-aDO_2_ over time is shown in Figure 2. Moreover, the results of the regression models showed no significant differences between groups in any measured clinical variable including fluid balance, urine output, and weight gain. Mean duration of mechanical ventilation was 6.3 (±4), 5.65 (±4) and 4.64 (±4) days in UE, E and S groups respectively (p > 0.05). Similarly, although PICU LOS was on average 2 days less in group S than the other groups (9.17 vs. 11.24 and 11.82 days, respectively, p > 0.05), this difference was not significant when logistic regression accounted for the inter-group differences in age, RACHS and CPB time. The inotrope score during the first 72 hours post-surgery was lower in group S compared to the other groups (Table 2). One death occurred in each group.

**Figure 3:**
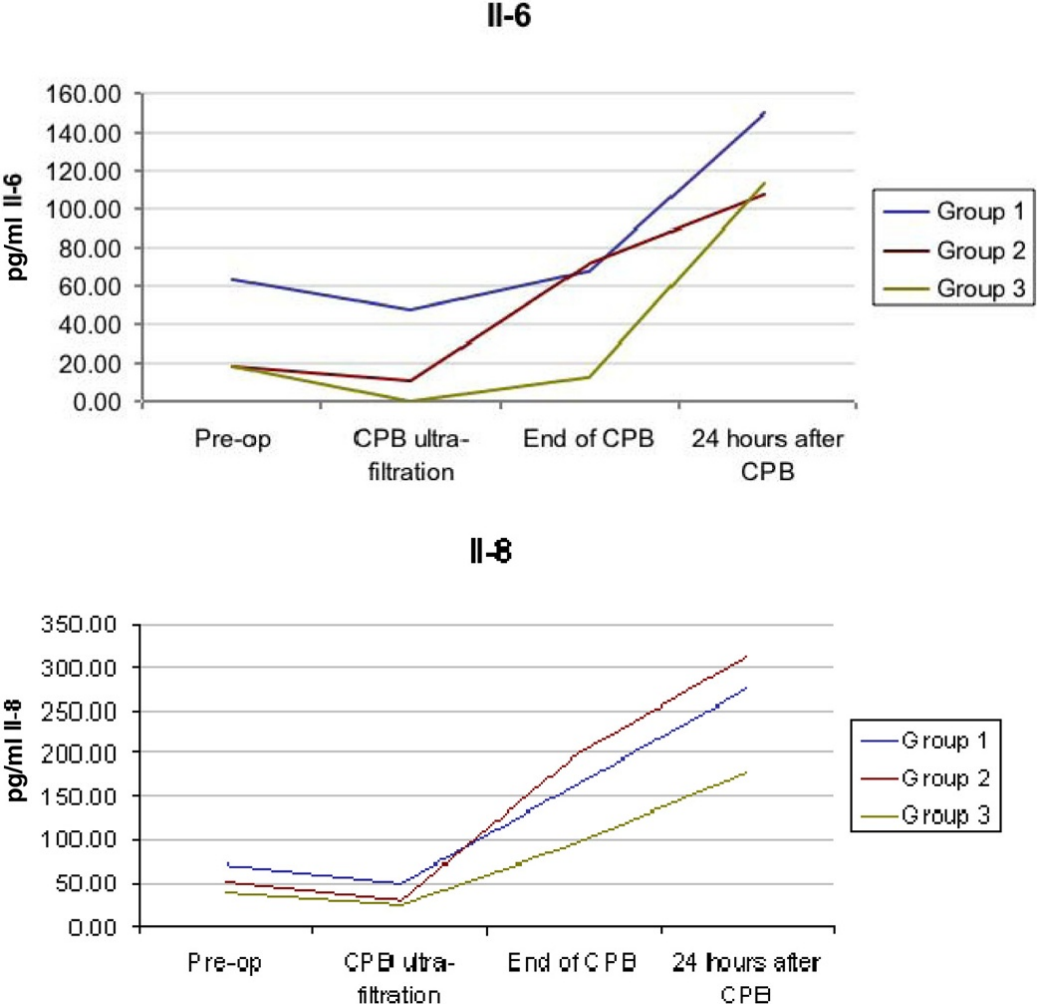
**Evolution of mean IL-6 and IL-8 over time stratified by study group.**

Inflammatory mediator and glutathione ratio data were collected for 51 patients (94%). Baseline IL-6 values were higher in group UE (63.61 ± 101.36 pg/ml) than group E (18.27 ± 22.42 pg/ml) or group S (17.96 ± 34.16 pg/ml) and rose higher than in these groups (Figure 3). All 3 groups had similar baseline IL-8 values with no significant differences between the groups at any time point.

**Figure 4:**
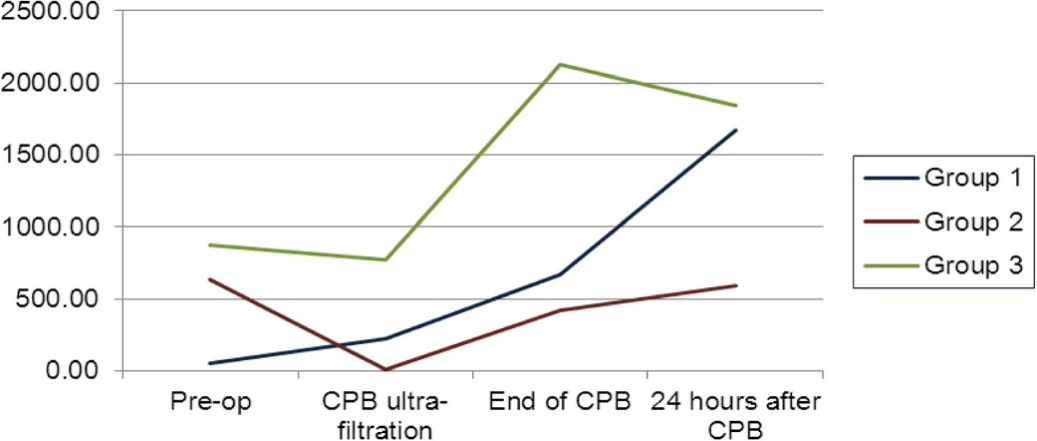
**Evolution of mean GSH:GSSG ratio over time stratified by study group.**

Regarding the evolution of the GSH:GSSG ratio, changes were most marked in group UE, which presented a delayed decrease in comparison to the other groups between ultra-filtration and end-CPB (Figure 4). Group E showed the least change with time with no significant difference between pre-operative and 24 hour post-op values, a different pattern from the other 2 groups which both showed increases from baseline. Furthermore, considering all time-points, overall GSH:GSSG ratio of group S was significantly higher (1179: 95% CI 25.05, 2332.75) than that of the other 2 groups.

**Table 2:** **Clinical outcomes stratified by study group**

Variable (mean)	Ultra-early group (n = 18)	Early group (n = 17)	Standard group (n = 19)	p*
**Weight (kg)**				
Pre-operative (SD)	3.73 (0.84)	3.65 (1.30)	4.60 (2.27)	>0.05
24 hours after surgery (SD)	4.20 (0.85)	4.23 (1.65)	4.74 (1.91)	>0.05
48 hours after surgery (SD)	4.15 (0.85)	4.10 (1.47)	5.10 (2.11)	>0.05
72 hours after surgery (SD)	4.00 (0.87)	3.94 (1.36)	5.06 (2.11)	>0.05
**Urinary output (ml/kg/h)**				
24 hours (SD)	1.47 (0.64)	1.27 (0.62)	1.36 (0.68)	>0.05
48 hours (SD)	4.41 (1.47)	4.58 (1.73)	3.43 (1.32)	0.047^δ^ and 0.037^Ψ^
72 hours (SD)	5.24 (1.69)	5.56 (2.08)	4.71 (1.31)	>0.05
**Balance (ml/kg/day)**				
24 hours (SD)	61.47 (39.48)	46.50 (40.36)	57.13 (70.43)	>0.05
48 hours (SD)	–34.66 (47.60)	-33.94 (48.73)	-11.63 (35.40)	>0.05
72 hours (SD)	-54.53 (47.80)	-53.50 (44.55)	-36.28 (31.20)	>0.05
**Inotrope score**				
24 hours (SD)	16.41 (6.39)	13.96 (14.86)	11.81 (25.11)	>0.05
48 hours (SD)	17.71 (6.23)	13.12 (10.27)	9.25 (24.68)	>0.05
72 hours (SD)	18.33 (5.05)	15.66 (6.83)	13.80 (24.58)	>0.05
**MV duration (days, SD)**	6.30 (3.51)	5.65 (4.18)	4.94 (3.62)	>0.05
**PICU LOS (days, SD)**	11.82 (11.77)	11.24 (9.10)	9.17 (5.65)	>0.05
**Death (%)**	1 (0.06)	1 (0.06)	1 (1.19)	>0.05

SD = standard deviation, MV = mechanical ventilation, PICU LOS = pediatric intensive care unit length of stay.

*Comparison between study groups (UE vs. S, UE vs. E, and E vs. S).

^δ^Comparison between UE and S groups.

^Ψ^Comparison between E and S groups.

## Discussion

Our findings do not support the hypothesis that a single ultra-early corticosteroid dose, compared to single doses at induction of anesthesia or on CPB, reduces the inflammatory response to CPB.

While there was no statistical difference in A-aDO2 between the groups in the present study, A-aDO2 was higher at 6 hours post-CPB in both early groups, and higher at 24 hours post-CPB in the UE group. Our results contradict the findings from previous published studies. However, all the animal and human studies that so far addressed this question (Lodge et al. 1999; Schroeder et al. 2003; Graham 2011; Clarizia et al. 2011) compared the use of double dose of steroids (early + prime) to prime administration only. Our aim was to determine if the previously described improvements in oxygenation were due to the earlier timing of the first steroid dose or to administration of two doses. The latter would appear to be the case.

The differences demonstrated by Lodge et al. (1999) in piglets were of markedly improved post-operative A-aDO_2_ gradient (absolute values not provided; p < 0.0003), pulmonary hemodynamics (p = 0.036), lung water (86.4%vs. 82.9%; p = 0.001) and body weight gain (96 g/kg vs. 160 g/kg; p) in the 8 and 1.5 hour pre-op-double dose corticosteroid group compared to placebo. Those who received methylprednisolone in the prime alone had results intermediate between placebo and the pre-op/double dose groups.

The retrospective review of Clarizia et al. (2011) also demonstrated better outcomes in infants receiving 2 versus 1 doses of steroid. Similar to the study of Lodge et al. (1999), they observed that patients receiving a single intraoperative dose (30 mg/kg methylprednisolone) had outcomes between those receiving two pre-operative doses (10 mg/kg on pre-operative evening, 10 mg/kg 2 hours pre-induction) plus the intraoperative dose and controls (duration of mechanical ventilation 4.4 vs. 3.2 vs. 5 days, respectively; p = 0.002). The RCT of Schroeder et al. (2003) evaluated the use of an early dose (4 hours pre-CPB) plus a prime dose of steroids in infants versus a single prime dose. They showed improvement in the arterio-venous oxygen gradient at 24 hours, as well as in PICU LOS and incidence of low-cardiac output syndrome (LCOS) in their double-dose group. Finally, Graham (2011) observed lower plasma levels of IL-6 after the pre-operative dose of corticosteroids, but no significant difference in the incidence of LCOS between neonates who received methylprednisolone in the pump prime only and those who also had a dose 8 hours pre-CPB and in the prime. Renal function was worse in the double-dose group.

Oxidative stress has rarely been studied in pediatric CPB. Two animal studies have shown increased tissue anti-oxidant activity after methylprednisolone given 24 hours to 5 days pre-CPB (Valen et al. 2000a, [b]). Christen et al. noted that oxidative stress preceded the peak in the inflammatory response (Christen et al. 2005). It is interesting to note in the present study that the whole blood GSH:GSSG ratio was highest in the S group, suggesting less oxidative stress. Further, this ratio was low pre-operatively and during CPB, rising afterwards, compatible with oxidative stress occurring early on during CPB.

Our baseline levels of IL-6 and IL-8 are significantly higher than those described by some pediatric cardiac studies, where mean values range from zero to 20 pg/ml (Schroeder et al. 2003; Graham 2011; Christen et al. 2005; Ashraf et al. 1997a), however others (Jonas 2004) have quoted much higher baseline values (~200 pg/ml). Our higher levels from baseline sampling the day before surgery may be related to a sicker population some of whom had undergone atrial septostomies. However the peak values that we obtained are similar or smaller in magnitude to those reported by multiple studies (Hauser et al. 1998; Schroeder et al. 2003; Santos et al. 2007).

The cytokine pattern differed between the three groups. IL-6 rose rapidly in the UE and S groups following the end of CPB, while the E group rose during CPB, and continued rising afterwards. All three groups had a rise in IL-8 during and following CPB. This is compatible with the work of Christen et al. (2005) in humans showing that the inflammatory response lagged behind oxidative stress as measured by ascorbate and malondialdehyde. It is important to highlight that the rise in IL-8 was less in the S group compared to the UE and E groups which may be due to less oxidative stress in this group.

Modification of cytokine release during pediatric CPB has been attempted by a variety of physical and biochemical methods (Allan et al. 2010; Ashraf et al. 1997b; Horton et al. 1999; Finn et al. 1996; Gessler et al. 2005; Sekido et al. 1993). These strategies have been largely unsuccessful and expensive. Corticosteroids have the advantage of being inexpensive, widely available and have been employed to attenuate the inflammatory response to CPB for decades. Schroeder et al. (Schroeder et al. 2003) showed that the plasma levels of IL-6 were lower by the end of CPB in the double-dose group (p < 0.05), but IL-6 values at 24 hours did not differ between the groups. Graham (2011) reported that the use of a double dose of corticosteroids reduces pre-operative IL-6, but both double-dose and at prime groups had a similar pattern and magnitude of increase in IL-6 in the first 24 hours post-operatively. However improvement in clinical outcomes remains the primary goal and has not been uniformly correlated with changes in cytokine release (Graham 2011; Keski-Nisula et al. 2013).

Because none of the studies discussed above used a single early dose of corticosteroids as their intervention, it is possible that the positive results shown by them (Lodge et al. 1999; Schroeder et al. 2003; Graham 2011; Clarizia et al. 2011) are due to the administration of two doses of steroids and not to their early administration, which left the question regarding the effect of timing unanswered. Our study is the first to compare different timings of one corticosteroid dose alone.

The main limitation of our study is the unbalanced randomisation of our cases with respect to age and complexity of cardiac disease (RACHS). Nevertheless, the use of random effects models allowed adjustment for these variables and also accounted for repeated measures of different variables per patient. Moreover, although the RACHS was statistically significantly different, a review of the actual lesions along with CPB and cross-clamp times reveal very little difference between the ultra-early and early groups, despite which the former group spent longer times ventilated and on the PICU. Our sample size calculation was based on Lodge et al., who demonstrated a very large difference between groups (Lodge et al. 1999). However, if this difference is actually smaller, we were underpowered to observe it. In addition, due to the small number of cases who received aprotinin, we could not evaluate its effect as a modulator of cytokine release during CPB. Some authors have also suggested a modulating effect of tranexamic acid on inflammatory response however this appears to be much less than that of aprotinin and has not been clearly demonstrated in infants (Graham et al. 2012; Later et al. 2009). Finally, this was a single institution study, which lead to a long duration of recruitment. However there were no major changes in anesthesic technique, management of CPB or post-operative care during this period and the same surgeon performed all the operations.

## Conclusions

In conclusion we observed that the timing of a single methylprednisolone dose alters neither AaDO_2_ at 72 hours nor clinical variables, including duration of mechanical ventilation in infants undergoing corrective congenital heart surgery. While standard clinical parameters did not differ between the groups, the standard group required less inotropic support, had a shorter length of stay, and less oxidation of glutathione. Generally the ultra-early group had more evidence of inflammation and oxidative stress. Although this group were younger and had higher RACHS Scores statistical analysis of the results suggest that the standard approach of administering corticosteroids at the start of bypass, rather than prior to surgery, may be the better approach. A larger multi-centered study is required to verify these findings.
